# Pharmacogenetics of amfepramone in healthy Mexican subjects reveals potential markers for tailoring pharmacotherapy of obesity: results of a randomised trial

**DOI:** 10.1038/s41598-019-54436-z

**Published:** 2019-11-28

**Authors:** Magdalena Gómez-Silva, Everardo Piñeyro-Garza, Rigoberto Vargas-Zapata, María Elena Gamino-Peña, Armando León-García, Mario Bermúdez de León, Adrián Llerena, Rafael B. R. León-Cachón

**Affiliations:** 1 Forensic Medicine Service, School of Medicine, Autonomous University of Nuevo Leon, Monterrey, Nuevo Leon Mexico; 2 Analytical Department of the Research Institute for Clinical and Experimental Pharmacology, Ipharma S.A, Monterrey, Nuevo Leon Mexico; 3 Clinical Department of the Research Institute for Clinical and Experimental Pharmacology, Ipharma S.A, Monterrey, Nuevo Leon Mexico; 4 Quality Assurance Department of the Research Institute for Clinical and Experimental Pharmacology, Ipharma S.A, Monterrey, Nuevo Leon Mexico; 5 Statistical Department of the Research Institute for Clinical and Experimental Pharmacology, Ipharma S.A, Monterrey, Nuevo Leon Mexico; 6 Pharmaceutical Research S.A, Mexico City, CDMX Mexico; 7 Department of Molecular Biology, Center for Biomedical Research of the Northeast, Mexican Institute of Social Security, Monterrey, Nuevo Leon Mexico; 8 Clinical Research Center of Health Area, Hospital and Medical School of Extremadura University, Badajoz, Spain; 9 Center of Molecular Diagnostics and Personalized Medicine, Department of Basic Sciences, Division of Health Sciences, University of Monterrey, San Pedro Garza Garcia, Nuevo Leon Mexico

**Keywords:** Genetic testing, Predictive markers

## Abstract

Amfepramone (AFP) is an appetite-suppressant drug used in the treatment of obesity. Nonetheless, studies on interindividual pharmacokinetic variability and its association with genetic variants are limited. We employed a pharmacokinetic and pharmacogenetic approach to determine possible metabolic phenotypes of AFP and identify genetic markers that could affect the pharmacokinetic variability in a Mexican population. A controlled, randomized, crossover, single-blind, two-treatment, two-period, and two sequence clinical study of AFP (a single 75 mg dose) was conducted in 36 healthy Mexican volunteers who fulfilled the study requirements. Amfepramone plasma levels were measured using high-performance liquid chromatography mass spectrometry. Genotyping was performed using real-time PCR with TaqMan probes. Four AFP metabolizer phenotypes were found in our population: slow, normal, intermediate, and fast. Additionally, two gene polymorphisms, *ABCB1*-rs1045642 and *CYP3A4*-rs2242480, had a significant effect on AFP pharmacokinetics (*P* < 0.05) and were the predictor factors in a log-linear regression model. The *ABCB1* and *CYP3A4* gene polymorphisms were associated with a fast metabolizer phenotype. These results suggest that metabolism of AFP in the Mexican population is variable. In addition, the genetic variants *ABCB1*-rs1045642 and *CYP3A4*-rs2242480 may partially explain the AFP pharmacokinetic variability.

## Introduction

Currently, obesity is a major health problem worldwide. According to the organization for economic co-operation and development (OECD), in 2017, Mexico was among the countries with the highest obesity rates worldwide^1^. Today, 36.3% of adolescents aged 12–19 years, and 72.5% of adults are overweight or obese^2^. Strictly, obesity is abnormal fat accumulation that can compromise health and be associated with other clinical conditions such as cardiovascular diseases, diabetes, musculoskeletal disorders, and cancer^3^.

Obesity is mainly caused by an energy surplus between calories consumed and calories expended^3^. Therefore, most treatments are focused on calorie-restricted diets and an increase in physical activity. Only when aforementioned treatments yield unsatisfactory results, pharmacological treatment is recommended^4,5^. Anti-obesity pharmacotherapy is limited to a few options that either suppress appetite or increase energy expenditure^4^. At present, the Food and Drug Administration (FDA) has approved the short-term use of appetite suppressants such as amfepramone (AFP) and phentermine (PHE) to treat obesity^5–7^; however, adverse drug reactions have been reported for some of them^4,8,9^.

The drug AFP, is classified as a schedule IV controlled substance in the United States and Canada because it acts on the central nervous system^10^, where it not only induces the release of the neurotransmitters noradrenaline (NA) and dopamine (DA) but also suppresses their reuptake. This mechanism of action results in less appetite^11,12^. However, owing to the psychostimulant effects of AFP, cautions should be taken regarding the dose and in patients with a psychiatric or cardiovascular history^13–15^. Specifically, in a Mexican population, AFP has been proven to be effective and safe for long-term treatment^16^. Nonetheless, some mild to moderate adverse events have been associated with AFP use, such as dry mouth, insomnia, anxiety, irritability, constipation, headache, dizziness, and polydipsia^12,13,16^.

The wide range of pharmacological effects of drugs, from being safe and effective to the risk of adverse reactions, may involve genetic factors that control absorption, distribution, metabolism, and excretion (ADME) processes^17,18^ and, therefore, pharmacokinetic parameters^19^. For example, recent studies in a Mexican population have shown that genetic variation may explain 30–90% of the pharmacokinetic variability^20,21^ of Statins. Here, the most common genetic variation responsible for these pharmacokinetic discrepancies are polymorphisms in genes encoding metabolizing enzymes and transporters^19–21^. Nevertheless, no pharmacogenetic data is available for AFP.

Therefore, to date, although after oral administration, AFP is extensively biotransformed to secondary metabolites; until now, the responsible metabolic enzymes are unknown^22–24^. Two metabolic pathways seem to be involved: 1) N-dealkylation and reduction^22,23^ and 2) N-deethylation^24^. The enzymes encoded by *CYP3A4* and *CYP3A5* are the most important because they metabolize a significant percentage of drugs. These enzyme isoforms have similar substrate selectivity, and therefore metabolize the same compounds^25^. Previous studies reported that CYP3A4 performs N-dealkylation and N-deethylation^26,27^. Therefore, CYP3A4 and CYP3A5 may have an important role in AFP metabolism. However, other CYP isoforms^25^ and other enzymes of phase II metabolism, such as glutathione S-transferases (GSTs), may also be involved^28^.

Amfepramone and its metabolites are excreted via the kidney^22–24^. Transporter proteins known to be involved in drug removal, such as the ones encoded by *ABCB1*, *ABCG2*, and *SLCO1B1*, may affect AFP plasma levels in the Mexican population^20,21^.

Because variations in genes involved in the ADME processes may affect the pharmacokinetic profile and, therefore, the effectivity of a drug, the aims of this study were: (1) to classify the AFP metabolism, (2) to evaluate the impact of genetic polymorphism related to drug metabolism in the Mexican population on AFP pharmacokinetics, and (3) to assess a possible association between genotypes and metabolizer phenotypes.

## Results

### Study population

A total of 36 unrelated healthy volunteers, aged between 18 and 51 years, of either sex and of Mexican origin, were enrolled and treated with AFP. Demographical, clinical, and pharmacokinetic data are presented in Table 1. Despite the fact that height and weight values were significantly higher in males than in females (*P* ≤ 6.97 × 10^−5^); the Body Mass Index (BMI) was not significantly different. No other features displayed significant variability (Table 1).

**Table 1:** Demographic data and pharmacokinetic descriptive statistics of volunteers.

n	All subjects	Males	Females
	36	19	17
Age (years)	24.25 ± 6.35	25.74 ± 8.25	22.59 ± 2.48
BMI (kg/m^2^)	23.81 ± 2.16	24.19 ± 1.96	23.39 ± 2.36
Height (m)	1.67 ± 0.10	1.74 ± 0.08^a^	1.59 ± 0.06
Weight (kg)	66.57 ± 11.23	73.12 ± 9.58^b^	59.25 ± 8.05
C_max_ (ng/mL)	7.04 ± 2.86	7.40 ± 3.46	6.64 ± 2.01
AUC_0-t_ (ng/mL/h)	29.87 ± 12.40	30.91 ± 15.57	28.72 ± 7.79
AUC_0-∞_ (ng/mL/h)	36.59 ± 13.66	38.38 ± 17.40	34.55 ± 7.71
K_e_	0.1923 ± 0.0539	0.2056 ± 0.0637	0.1774 ± 0.0365
T_1/2_ (h)	3.95 ± 1.45	3.84 ± 1.83	4.07 ± 0.91
Cl (L/h/kg)	2.32 ± 0.90	2.37 ± 1.16	2.27 ± 0.51
V_d_ (L/kg)	12.92 ± 5.47	12.47 ± 6.38	13.42 ± 4.38

Data shown as mean ± standard deviation. ^a^*P* = 5.24 × 10^−6^; ^b^*P* = 6.97 × 10^−5^. *BMI*, body mass index; *C*_*max*_, maximum plasma concentration; *AUC*, area under the plasma concentration-time curve; *AUC*_*0-t*_, AUC from time 0 to the time of last measurement; *AUC*_*0-∞*_, AUC from time 0 extrapolated to infinity; *K*_*e*_, elimination rate constant in the terminal drug phase; *T*_*1/2*_, half-life drug; *V*_*d*_, volume of distribution; *Cl*, total drug clearance.

### Amfepramone pharmacokinetics

The mean ± SD values for the pharmacokinetic parameters for all subjects were as follows: maximum plasma concentration (C_max_) = 7.04 ± 2.86 ng/mL, area under the plasma concentration-time curve from 0 to the time of last measurement (AUC_0-t_) = 29.87 ± 12.39 ng/mL/h, AUC from time 0 extrapolated to infinity (AUC_0-∞_) = 36.59 ± 13.66 ng/mL/h, elimination rate constant in the terminal drug phase (Ke) = 0.1923 ± 0.0539, half-life (T_1/2_) = 7.04 ± 2.86 h, total drug clearance (Cl) = 2.32 ± 0.90 L/h/kg, volume of distribution (Vd) = 7.04 ± 2.86 L/kg. No sex differences were found in pharmacokinetic parameters (Table 1). Despite the lack of significant differences in the demographic and clinical features (Table 1), there was a remarkable pharmacokinetic variability (Table 2).

**Table 2:** Pharmacokinetic parameters according to metabolizer phenotype.

Pharmacokinetic parameters	Metabolizer phenotypes for all subjects			
	Slow	Intermediate	Normal	Fast
N	7	9	12	8
C_max_ (ng/mL)	11.07 ± 3.49^a^	7.21 ± 0.91^a^	6.51 ± 1.16^a^	4.12 ± 0.89^a^
AUC_0-t_ (ng/mL/h)	45.84 ± 14.69^b^	34.98 ± 2.13^b^	26.44 ± 2.87^b^	15.30 ± 3.74^b^
AUC_0-∞_ (ng/mL/h)	51.61 ± 15.59^c^	43.17 ± 10.58^c^	32.87 ± 2.96^c^	21.62 ± 4.67^c^
K_e_	0.2013 ± 0.0431	0.2063 ± 0.0708	0.1794 ± 0.0452	0.1880 ± 0.0577
T_1/2_ (h)	3.58 ± 0.80	4.01 ± 1.15	4.11 ± 1.15	3.96 ± 1.08
Cl (L/h/kg)	1.53 ± 0.32^d^	1.80 ± 0.31^d^	2.30 ± 0.20^d^	3.64 ± 0.91^d^
V_d_ (L/kg)	7.89 ± 2.13^e^	9.59 ± 2.87^e^	13.68 ± 4.08^e^	19.90 ± 3.75^e^

Data shown as mean ± standard deviation. ^a^*P* ≤ 9.37 × 10^−3^; ^b^*P* ≤ 0.001; ^c^*P* ≤ 0.023; ^d^*P* ≤ 0.026; ^e^*P* ≤ 0.002; ^c^*P* ≤ 9.79 × 10^−6^. *C*_*max*_, maximum plasma concentration; *AUC*, area under the plasma concentration-time curve; *AUC*_*0-t*_, AUC from time 0 to the time of last measurement; *AUC*_*0-∞*_, AUC from time 0 extrapolated to infinity; *K*_*e*_, elimination rate constant in the terminal drug phase; *T*_*1/2*_, half-life drug; *V*_*d*_, volume of distribution; *Cl*, total drug clearance.

### Metabolic phenotype classification

**Figure 1:**
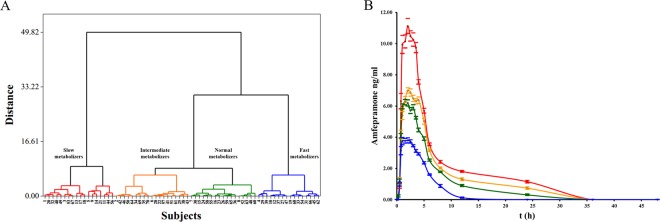
Classification of amfepramone (AFP) metabolic phenotypes. (**A)** Dendrogram generated with Manhattan distance and Ward’s linkage method. (**B)** Pharmacokinetic profiles of different metabolic phenotypes. Mean peak plasma AFP concentration-time curves after single 75 mg dose of AFP. Data shown are mean ± standard error (SE) concentrations. For both (**A**,**B)**: slow metabolizers (*red*), intermediate metabolizers (*orange*), normal metabolizers (*green*), and fast metabolizers (*blue*).

Based on the multivariate analysis^20,29–31^ of the parameters C_max_ and AUC_0-t_, we identified four AFP phenotypes–slow metabolizers (n = 7), intermediate metabolizers (n = 9), normal metabolizers (n = 12), and fast metabolizers (n = 8)–as shown in Fig. 1A. The means of most pharmacokinetic parameters were significantly different among the four metabolizer phenotypes (*P* ≤ 0.026, Table 2). We used AUC_0-t_ values to validate the pharmacokinetic variability. The regression analysis suggests that the four metabolizer phenotypes offer a better prediction of the variability of AUC_0-t_ (R = 0.888, R^2^ = 0.788, adjusted R = 0.781, *P* = 5.50 × 10^−13^; AICC = 144.02; and ASE = 7.15). There was a > 6-fold difference in AFP pharmacokinetic parameters between the slowest metabolizer (C_max_ = 17.80 ng/mL and AUC_0-t_ = 78.85 ng/mL/h) and the fastest metabolizer (C_max_ = 2.94 ng/mL and AUC_0-t_ = 10.31 ng/mL/h). The pharmacokinetic profiles of the different metabolizer phenotypes are presented in Fig. 1B.

### Pharmacogenetic tests

All gene polymorphisms were in Hardy Weinberg Equilibrium (HWE) (*P* > 0.05) and passed the quality control tests of genotyping. Genotype frequencies are presented in Table 3.

**Table 3:** Polymorphisms with significant effect on AFP pharmacokinetics.

Genotypes	n	Pharmacokinetics parameters						
		C_max_ (ng/mL)	AUC_0-t_ (ng/mL/h)	AUC_0-∞_ (ng/mL/h)	K_e_	T_1/2_ (h)	Cl (L/h/kg)	V_d_ (L/kg)
***ABCB1*-rs1045642**								
C/C	10	5.42 ± 1.88^a^	23.29 ± 9.57	29.02 ± 9.74	0.2193 ± 0.0533^b^	3.35 ± 0.90	2.94 ± 1.22	13.85 ± 5.13
C/T	13	8.15 ± 3.63	34.36 ± 15.42	42.10 ± 17.64	0.1901 ± 0.0628	4.20 ± 2.05	2.01 ± 0.63	11.85 ± 5.67
T/T	13	7.18 ± 2.11	30.45 ± 9.13	36.91 ± 9.01	0.1738 ± 0.0375	4.16 ± 0.95	2.17 ± 0.62	13.26 ± 5.78
C/T + T/T	26	7.66 ± 2.95^c^	32.40 ± 12.57^d^	39.50 ± 13.97^e^	0.1819 ± 0.0513	4.18 ± 1.57	2.09 ± 0.62	12.56 ± 5.65
***ABCG2*-rs2231142**								
C/C	25	6.77 ± 2.33	28.05 ± 9.10	35.04 ± 11.26	0.1928 ± 0.0577	4.01 ± 1.67	2.39 ± 0.94	13.26 ± 5.43
C/A	11	7.66 ± 3.87	34.01 ± 17.66	40.11 ± 18.14	0.1911 ± 0.0462	3.79 ± 0.84	2.17 ± 0.83	12.14 ± 5.75
***SLCO1B1*-rs4149056**								
T/T	32	7.09 ± 2.95	30.26 ± 12.84	37.01 ± 14.30	0.1959 ± 0.0553	3.90 ± 1.51	2.32 ± 0.95	12.68 ± 5.58
C/T	4	6.65 ± 2.26	26.75 ± 8.62	33.26 ± 6.84	0.1638 ± 0.0323	4.35 ± 0.84	2.32 ± 0.41	14.79 ± 4.75
***DRD3*-rs6280**								
C/C	8	7.10 ± 1.71	29.77 ± 8.09	35.48 ± 9.10	0.1994 ± 0.0521	3.69 ± 0.98	2.22 ± 0.51	11.73 ± 3.66
C/T	19	7.11 ± 3.38	30.47 ± 15.03	36.49 ± 14.91	0.1959 ± 0.0499	3.76 ± 1.00	2.37 ± 1.01	13.01 ± 6.42
T/T	9	6.85 ± 2.73	28.71 ± 10.20	37.78 ± 15.51	0.1784 ± 0.0665	4.58 ± 2.36	2.31 ± 1.01	13.76 ± 4.94
***CYP3A4*-rs2242480**								
C/C	13	8.49 ± 3.86	34.77 ± 15.64 ^f^	40.38 ± 16.27	0.1993 ± 0.0454	3.63 ± 0.76	2.10 ± 0.74	11.12 ± 4.88
C/T	14	5.52 ± 1.62 ^g^	24.60 ± 9.68	32.42 ± 13.77	0.1924 ± 0.0688	4.21 ± 2.07	2.70 ± 1.15	15.05 ± 5.90
T/T	9	7.32 ± 1.25	30.99 ± 8.00	37.60 ± 7.50	0.1821 ± 0.0412	4.00 ± 1.06	2.06 ± 0.39	12.20 ± 4.99
C/C + T/T	22	8.01 ± 3.08 ^h^	33.23 ± 12.95^i^	39.24 ± 36.59	0.1923 ± 0.0436	3.78 ± 0.89	2.08 ± 0.61	11.56 ± 4.84
***CYP3A5*-rs776746**								
A/A	1	6.30 ± NA	27.00 ± NA	34.56 ± NA	0.1500 ± NA	4.62 ± NA	2.17 ± NA	14.47 ± NA
A/G	13	6.44 ± 2.05	28.21 ± 11.55	36.18 ± 15.16	0.1913 ± 0.0605	4.16 ± 2.06	2.51 ± 1.27	13.73 ± 6.34
G/G	22	7.43 ± 3.29	30.99 ± 13.28	36.92 ± 13.40	0.1948 ± 0.0514	3.79 ± 1.01	2.22 ± 0.63	12.37 ± 5.11
***CYP2B6*-rs3745274**								
G/G	14	7.19 ± 3.66	32.17 ± 16.50	38.09 ± 16.16	0.2029 ± 0.0560	3.69 ± 1.14	2.26 ± 0.90	12.28 ± 6.57
G/T	18	6.84 ± 2.46	27.21 ± 8.88	34.69 ± 12.80	0.1845 ± 0.0545	4.18 ± 1.75	2.46 ± 0.98	13.88 ± 4.79
T/T	4	7.43 ± 1.57	33.80 ± 8.84	39.90 ± 8.30	0.1905 ± 0.0504	3.82 ± 0.98	2.32 ± 0.90	10.83 ± 4.35
***GSTM3*-rs1799735**								
*A/*A	29	7.18 ± 3.12	29.62 ± 13.59	36.66 ± 15.03	0.1922 ± 0.0572	4.00 ± 1.58	2.37 ± 0.98	13.26 ± 5.80
*A/*B	7	6.46 ± 1.28	30.93 ± 5.71	36.28 ± 5.78	0.1929 ± 0.0409	3.72 ± 0.75	2.12 ± 0.40	11.48 ± 3.83

Data shown as mean ± standard deviation. ^a^*P* = 0.042 (C/C *vs*. T/T); ^b^*P* = 0.046 (C/C *vs*. T/T); ^c^*P* = 0.031 (C/C *vs*. C/T + T/T); ^d^*P* = 0.041 (C/C *vs*. C/T + T/T); ^e^*P* = 0.037 (C/C *vs*. C/T + T/T); ^f^*P* = 0.033 (C/C *vs*. C/T); ^g^*P* = 0.021 (C/T *vs*. T/T); ^h^*P* = 0.005 (C/T *vs*. C/C + T/T); ^i^*P* = 0.038 (C/T *vs*. C/C + C/T). *C*_*max*_, maximum plasma concentration; *AUC*, area under the plasma concentration-time curve; *AUC*_*0-t*_, AUC from time 0 to the time of last measurement; *AUC*_*0-∞*_, AUC from time 0 extrapolated to infinity; *Ke*, elimination rate constant in the terminal drug phase; *T*_*1/2*_, half-life drug; *V*_*d*_, volume of distribution; *Cl*, total drug clearance.

### Association between genotypes and Amfepramone pharmacokinetics

Two of the analysed polymorphisms affected AFP pharmacokinetics. C_max_, AUC_0-t_, AUC_0-∞_, and Ke were affected by *ABCB1*-rs1045642 and *CYP3A4*-rs2242480 under the co-dominant, dominant, and over-dominant models (Tables 3 and 4). For *ABCB1*-rs1045642, the values of C_max_, AUC_0-t_, and AUC_0-∞_ in carriers of genotype C/C were significantly lower than those in carriers of the variant allele (T/T or C/T + T/T, *P* ≤ 0.042; Table 3), but the values of Ke (*P* = 0.046) in carriers of genotype C/C were significantly higher than those in carriers of the homozygous variant allele (T/T). The regression analysis confirmed our findings. Regarding *CYP3A4*-rs2242480, AUC_0-t_ was significantly lower in carriers of the heterozygous genotype (C/T) than in subjects with the C/C genotype (*P* = 0.033). Moreover, the C/T genotype was associated with lower C_max_ values than the T/T genotype (*P* = 0.021). In addition, the C_max_ and AUC_0-t_ values of the C/T genotype were significantly lower than those in the combined homozygous subjects (C/C + T/T; *P* ≤ 0.038; Table 3).

**Table 4:** Analysis of predictor models for the pharmacokinetic parameters of amfepramone.

Parameter	Predictors	Genetic model	R square	Adjusted R square	*P*-Value
K_e_	rs1045642	Co-dominant	0.113	0.087	0.045*
C_max_	rs1045642	Dominant	0.173	0.149	0.012*
AUC_0-t_	rs1045642	Dominant	0.143	0.117	0.023*
AUC_0-∞_	rs1045642	Dominant	0.163	0.139	0.037*
Cl	rs1045642	Dominant	0.185	0.161	0.009*
C_max_	rs224280	Over-dominant	0.223	0.200	0.004*
AUC_0-t_	rs224280	Over-dominant	0.146	0.121	0.021*
C_max_	rs224280, rs1045642	Over-dominant, dominant	0.248	0.202	0.009*
AUC_0-t_	rs224280, rs1045642	Over-dominant, dominant	0.181	0.132	0.037*
Cl	rs224280, rs1045642	Over-dominant, dominant	0.240	0.194	0.011*

*P-Value supported by automatic linear modeling and log-linear regression analysis and method. *C*_*max*_, maximum plasma concentration; *AUC*, area under the plasma concentration-time curve; *AUC*_*0-t*_, AUC from time 0 to the time of last measurement; *AUC*_*0-∞*_, AUC from time 0 extrapolated to infinity; *Ke*, elimination rate constant in the terminal drug phase; *Cl*, total drug clearance.

The importance of aforementioned variability in AFP pharmacokinetics was confirmed by a regression analysis. However, after this analysis, the effect of *ABCB1*-rs1045642 polymorphism on Cl was revealed using a dominant model (Table 4). The final prediction model included *ABCB1*-rs1045642 and *CYP3A4*-rs2242480 (*P* ≤ 0.037; Table 4).

### Association between genotypes and metabolizer phenotypes

The association between genotype and phenotype was evaluated under the various genetic models and combinations of genotypes. We found two polymorphisms associated with the fast metabolizer phenotype (Table 5). The C/C genotype of *ABCB1*-rs1045642 under the co-dominant and dominant model and the C/T genotype of *CYP3A4*-rs2242480 under the over-dominant model were significantly associated with the fast metabolizer phenotype (Table 5) after Bonferroni’s correction (*P* ≤ 0.049).

**Table 5:** Association between genotypes and metabolizer phenotypes

Gene	Polymorphism	Model	OR (95% CI)	*P*-Value	Pc-Value
*ABCB1*	rs1045642	Dominant (C/C vs. C/T + T/T)	C/C: Fast metabolizers	0.013	0.041*
			0.29 (0.11–0.75)		
			C/T + T/T: Slow/intermediate/normal metabolizers		
			2.19 (0.88–5.45)		
*CYP3A4*	rs224280	Co-dominant	C/T: Fast metabolizers	0.033	
*CYP3A4*	rs224280	Over-dominant (C/T vs. C/C + T/T)	C/C + T/T: slow metabolizers	0.019	0.055
			1.93 (1.36–2.75)		
*CYP3A4*	rs224280	Over-dominant (C/T vs. C/C + T/T)	C/T: Fast metabolizers	0.018	0.049*
			0.38 (0.19–0.77)		
			C/C + T/T: Slow/intermediate/normal metabolizers		
			2.86 (0.84–9.71)		
Combination	rs1045642, rs224280	Dominant (C/C vs. C/T + T/T), Over-dominant (C/T vs. C/C + T/T)	C/C-C/T: Fast metabolizers	0.004	0.014*
			0.14 (0.03–0.64)		
Combination	rs1045642, rs224280	Dominant (C/C vs. C/T + T/T), Over-dominant (C/T vs. C/C + T/T)	C/T + T/T-C/C + T/T: Slow metabolizers	0.003	0.012*
			2.64 (1.66–4.20)		

^*^P-Value supported by logistic regression analysis. *OR*, odds ratio; *CI*, confidence interval; *Pc*, Bonferroni-corrected P-values.

## Discussion

In Mexico, the issue of obesity is alarming because of its high prevalence^1^; however, only a small fraction of the population goes to professional health services for diagnosis and treatment^32^. Pérez-Salgado *et al*. suggest that this could be because the government institutions in Mexico focus mainly on raising awareness among the population about diseases that derive from obesity^32^ and, therefore, research focused on improving the effectiveness of treatments is needed.

In order to contribute to health needs, the pharmacokinetic variability of one of the drugs used in the treatment of obesity was tested in a Mexican population. In this study, AFP pharmacokinetic profiles of 36 healthy Mexican volunteers were assessed under controlled conditions. In our sample, the groups of men and women had similar age range and BMI and the same ethnic group. However, they were significantly different in terms of weight and height (Table 1). These differences that exist between men and women could affect the drug disposition^33^ and, therefore, influence the pharmacokinetic variability^34^. Nevertheless, this variability could be eliminated if normalization by weight is performed^33^. Accordingly, in our findings, no sex differences were observed in pharmacokinetic parameters among males and females (Table 1). To our knowledge, no prior studies have reported the effect of sex on AFP pharmacokinetics.

To verify whether there exists variation in AFP pharmacokinetic profiles and investigate a possible association with polymorphisms in genes related to drug metabolism, we used a simple approach to classify the pharmacokinetic profiles using the parameters C_max_ and AUC_0-t_. Four major metabolizer phenotypes were distinguished (slow, normal, intermediate, and fast) with significant differences in pharmacokinetic parameters among the metabolizer phenotype groups (Table 2, Fig. 1A,B). Although our metabolic classification should be validated by another phenotyping method such as pharmacometabolomics^35^, the pharmacokinetic variability among metabolic phenotypes is evident. This variability is confirmed by a >6-fold difference in the pharmacokinetic parameters between the slowest and the fastest metabolizers. To the best of our knowledge, this is the first report on different AFP metabolizer types. In 1973, Testa and Beckett reported minor intersubject variations under standardized conditions of AFP metabolism; however, their trials were performed on only four subjects^36^. During the clinical trial and at the end, no volunteers showed any adverse effects.

Although several pharmacogenetics and pharmacogenomics studies have been published, this is the first study evaluating AFP. In this study, two polymorphisms had a significant impact on AFP pharmacokinetics (*P* ≤ 0.046). One polymorphic variant involved a gene that encodes a transporter protein (*ABCB1*-rs1045642), and the other located polymorphism involved genes encoding drug-metabolizing enzymes (*CYP3A4*-rs2242480). It is known that *ABCB1* interacts with a multitude of structurally and biochemically unrelated substrates^37^. Therefore, *ABCB1* affects plasma concentrations of and, consequently, the pharmacological response to several drugs, with inconsistent results. The latter depends on the population as well as the substrate analyzed^20,37–44^, for example, in a Chinese study population, the T/T genotype was associated with lower plasma concentrations of clopidogrel and its active metabolites^45^. However, in Korean subjects, the T/T genotype was associated with higher C_max_ and AUC values of fexofenadine hydrochloride^46^. These discrepancies may be due to the fact that polymorphisms with silent effects can affect the time of protein translation and, therefore, its folding, causing changes in substrate specificity^47^. Regarding anti-obesity drugs, Lloret *et al*. conducted a study on the role of *ABCB1*-rs1045642 gene variant on oral morphine pharmacokinetics; however, they did not find an association between the two^48^. Nevertheless, there is no information on the effect of *ABCB1* on AFP pharmacokinetics. For the Mexican population, the C/C genotype of the rs1045642 polymorphism has been associated with the atorvastatin fast metabolizer phenotype because of a significant effect on C_max_ and AUC_0-t_ values among 60 healthy volunteers^20^. The results of the present study are consistent with the previous report; carriers of the C/C genotype showed significantly lower C_max_, AUC_0-t_, and AUC_0-∞_ values (Tables 3 and 4), significant variability in Ke and Cl values, and were associated with the AFP fast metabolizer phenotype after Bonferroni’s correction (Table 5). These results suggest that the effect of rs1045642 could be related to abnormal intestinal absorption and clearance of AFP. Here, the C/C genotype carriers showed higher Ke and Cl values but lower T_1/2_. However, only Ke was significantly different (Table 3). These findings are similar to those reported by Gonzalez-Vacarezza *et al*. in quetiapine pharmacokinetics^49^. To our knowledge, the present study is the first report on the effect of rs1045642 on AFP pharmacokinetics.

*CYP3A4* encodes one of the main drug metabolizing-enzymes^25^. Although the CYP3A4*1B polymorphic variant is the most studied, there is no report of an association between drug metabolism and response in the Mexican population^50–52^. Therefore, other polymorphic variants in non-coding regions have been explored, for example, *CYP3A4*-rs2242480 in intron 10^50^. Interestingly, we found that *CYP3A4*-rs2242480 affected several parameters of AFP pharmacokinetics (Tables 3 and 4). Individuals with the heterozygous genotype (C/T) showed a significant effect on C_max_ and AUC_0-t_ and a significant association with the fast metabolizer phenotype (Table 5), *i.e*. there is an increased capacity for plasma clearance. Nonetheless, our results suggest that the C/T genotype is associated with an increased biotransformation of AFP because rs2242480 significantly affects only C_max_ and AUC_0-t_ values but not parameters such as Ke and Cl (Tables 3 and 4). These results are consistent with the previous report in a Mexican population. The rs2242480 polymorphism was the most important variant of the prediction model of the variation of AUC values of atorvastatin^21^ and for the increased R-warfarin clearance in subjects with a heterozygous genotype in a population from the United Kingdom^53^. However, the present results differ from those reported by Danielak *et al*. in a Polish population where no effect of rs2242480 was observed on clopidogrel pharmacokinetics^50^. The T allele frequency (0.44) found in our study population was different from those reported in populations from the United Kingdom (0.09)^53^, Poland (0.08)^50^, and Asia (0.29)^54^.

In summary, AFP pharmacokinetics differs among Mexicans, and we found two genetic polymorphisms with a significant effect on AFP pharmacokinetics, which were used in a log-linear regression analysis. Nevertheless, the interaction of these polymorphisms could be considered to produce a small or moderate effect because only 13–20% of the variability on C_max_, AUC_0-t_, and Cl can be predicted (Table 4). It is possible that other gene polymorphisms not evaluated in this study may contribute to AFP pharmacokinetic variability.

Furthermore, we did not measure the concentration of secondary metabolites. Such information might have provided an additional phenotyping method to support our results. Another study limitation was the small population size. A follow-up study with a larger population is required to validate our results.

## Conclusion

This pilot study revealed variability in AFP pharmacokinetics in the Mexican population. Additionally, *ABCB1*-rs1045642 and *CYP3A4*-rs2242480 were found to significantly affect AFP plasma levels. These results suggest that genetic variants may be useful in studies predicting pharmacokinetic variability and those that evaluate the response to AFP treatment. Further studies in a larger population are required to validate our findings.

## Material and Methods

### Design

A controlled, randomized, crossover, single-blind, two-treatment, two-period, and two-sequence clinical study was conducted in healthy volunteers of Mexican origin to assess the bioequivalence of a single oral dose (75 mg) of AFP (extended-release capsule; Medix Products, S. A., Mexico City, CDMX, MEX) at Ipharma S.A. The study complies with the guidelines of the Declaration of Helsinki, the Good Clinical Practice Standards of Tokyo, and the Mexican regulations for studies of bioavailability and bioequivalence. The clinical protocol was approved by the Research and Ethics Committee of the Clinical and Experimental Pharmacology Center, Ipharma S. A. (Monterrey, NL, MEX), and the pharmacogenetic procedure was approved by the Ethics, Research, and Biosecurity Committees of the University of Monterrey (San Pedro Garza Garcia, NL, MEX; registry number 042014-CIE). The study was registered in the National Registry of Clinical Trials (RNEC; registry number /A394-16) at the Federal Commission for Protection Against Health Risks (COFEPRIS) and Australian New Zealand Clinical Trials Registry (ACTRN12619000391178; registration date: 12/03/2019). Written informed consent was obtained from all subjects or their parents/legal tutors.

### Study population

From February 2017 to March 2017, 36 healthy volunteers were enrolled and randomized using R statistical software version 2.15.2. Inclusion criteria for this study were as follows: healthy males and non-pregnant, non-breastfeeding females, aged 18–55 years, with a BMI in the range 18–27 kg/m^2^, non-smokers, and willing to use adequate methods of contraception throughout the study. Exclusion criteria included electrocardiographic, clinical, biochemical, haematological, coagulation, or urinalysis test abnormalities; a positive test for drug or alcohol abuse, human immunodeficiency virus, hepatitis B and C viruses, and rapid plasma reagin; a medical history of serious adverse reaction or serious hypersensitivity to the test drug or to chemically related drugs; glaucoma, hypertension, anorexia and thyroid problems or existence of concurrent disease; a history of smoking, alcohol or drug abuse; the use of prescription or over-the-counter medication 3 weeks before enrolment; and participation in a clinical research study in the previous 3 months.

### Randomization

The volunteers who fulfilled the requirements were selected by the clinical research coordinator of Ipharma, S.A. Before the start of the clinical study, the statistical division of Ipharma S.A. performed randomization for the allocation of drugs (R or P, file code, and subject code). The randomization was done in two blocks in a balanced design using the R statistical software version 2.15.2. and the Mersenne Twister algorithm. The participants were blinded to treatment allocation.

### Sampling

A single dose of 75 mg of AFP extended-release capsule (Medix Products, S. A., Mexico City, CDMX, MEX) was administered orally after overnight fasting. The volunteers were under medical supervision throughout the protocol. Blood samples (4 mL) were collected in BD K_2_EDTA-coated Vacutainers (BD Diagnostics, Franklin Lakes, NJ, USA) at pre-dose (time 0) and seventeen times after drug administration (0.25, 0.5, 0.75, 1, 1.25, 1.50, 1.75, 2, 2.5, 3, 4, 6, 8, 10, 12, 24, and 48 h). Plasma was separated by centrifugation (10 min at 1600 × g at 4 °C) and stored at −65 ± 15 °C for subsequent analysis.

### Determination of plasma Amfepramone concentration

Plasma AFP concentration was measured using a validated method developed by Ipharma S.A., according to Mexican regulations (NOM-177-SSA1-2013)^55^ and guidelines of European Medicines Agency (EMA) regarding bioanalytical method validation^56^.

In brief, the plasma proteins were eliminated by acetonitrile precipitation. Then, 300 µL acetonitrile was added to a 50 µL plasma sample, vortexed (60 rpm, 4 min), and centrifuged (9600 × *g*, 10 min, 10 °C). The supernatant (200 µL) was injected into a high-performance liquid chromatography (HPLC) system coupled to a tandem mass spectrometer 6410B (MS/MS) with a triple quadrupole detector (Agilent Technologies, Santa Clara, CA, USA). The chromatographic separation was performed at 45 °C with a Gemini C18 pre-column (Phenomenex, Torrance, CA, USA) and a Zorbax Eclipse XDB C-18 column (3.5 µm, 80 Å, 4.6 × 150 mm) (Agilent Technologies, Santa Clara, CA, USA). The injection sample volume was 5 µL. The mobile phase had a flow rate of 0.6 mL/min and consisted of 5.0 mM ammonium formate/0.1% formic acid and acetronitrile (15:85). The detection system used an ESI MS/MS precursor ion (+) 206.2 m/z and a (+) product ion 105.0 m/z. The interday linearity was assessed with calibration curves (curve range, 1 to 100 ng/mL: 1, 2.5, 5, 10, 25, 50 and 100 ng/mL). The intraday quality control was evaluated by control samples of 1.7, 7.5, 35, and 75 ng/mL each. The validated method had a coefficient of variation (CV) ≤5% for the precision and an error ≤5% for the accuracy.

### Pharmacokinetic analysis

The pharmacokinetic parameters were calculated by non-compartmental methods. The maximum plasma concentration (C_max_) and time to reach C_max_ (T_max_) were obtained from the concentration-time data of the plasma. The area under the plasma AFP concentration-time curve (AUC) from time 0 to the time of last measurement (AUC_0-t_), AUC from time 0 extrapolated to infinity (AUC_0-∞_) were calculated by log-linear trapezoidal rule. Ke was estimated by log-linear regression from the terminal portion of the log-transformed concentration-time plots. T_1/2_ was estimated by dividing 0.693 by Ke. The total drug clearance was calculated by dividing dose by AUC_0-∞_ and adjusting for weight. Volume of distribution was calculated as Cl divided by Ke. The AUC and C_max_ values were adjusted for dose and weight (AUC/dW and C_max_/dW). The pharmacokinetic analysis was performed using R statistical software version 2.15.2.

### Classification of Amfepramone metabolism

The metabolizer phenotypes were determined according to a multivariate analysis of the combined pharmacokinetic parameters C_max_ and AUC_0-t_ using a hierarchical cluster analysis (HCA)^20,29^. Since the clustering algorithms are dependent on the data size, we used a modified method to estimate the ideal number of clusters^30,31^. First, we introduced the data set of the unadjusted C_max_ and AUC_0-t_ values together with the adjusted data set. Second, C_max_ and AUC_0-t_ were standardized to minimize the effect of scale differences, and a distance matrix was made from the combined C_max_ and AUC_0-t_ values. Third, hierarchical cluster analysis (HCA) using the Ward linkage method was performed on individual C_max_ and AUC_0-t_ values. Finally, the interindividual Manhattan distances were computed. Minitab 16 software (Minitab Inc., State College, PA, USA) was used for standardization and HCA. We identified the subjects of each cluster and calculated the means of all adjusted pharmacokinetic parameters by cluster. According to the mean values of the pharmacokinetic parameters of the clusters, they were classified into metabolizer phenotypes^20^.

### Pharmacogenetic tests

The Wizard Genomic DNA Purification kit (Promega Corp., Madison, WI, USA) was used to isolate DNA according to manufacturer’s instructions. Genomic DNA was quantified by UV absorbance using Nanodrop (Thermo Fisher Scientific Inc., Wilmington, MA, USA). The DNA purity was evaluated with the A260/280 and A260/230 ratios, and the samples were stored at −20 °C until use. The DNA samples were subjected to genotyping for the polymorphisms *ABCB1*-rs1045642, *ABCG2*-rs2231142, *SLCO1B1*-rs4149056, *DRD3*-rs6280, *CYP3A4*-rs2242480, *CYP3A5*-rs776746, *CYP2B6*-rs3745274, and *GSTM3*-rs1799735^57^ using real-time polymerase chain reaction and Taqman probes (Applied Biosystems; Thermo Fisher Scientific Inc., Wilmington, MA, USA) according to the manufacturer’s protocol. Three quality control thresholds were applied: a genotype call rate equal to 1, an HWE test with *P* > 0.05, and minor allele frequency >0.01.

### Statistical analysis

For sample size calculation, the intrasubject CV obtained in a previous pilot study (Amfepramone/A359-16P) was considered. It was assumed that CV was 25.77% for both C_max_ and AUC. Considering a confidence level of 95%, a significance level of 5%, and a minimum power of 80%, a sample size of 30 would suffice. One-way analysis of variance (ANOVA); Kruskal Wallis test; automatic linear modelling using forward stepwise method, Akaike Information Criterion (AICC) and Overfit Prevention Criterion (ASE); and linear and logistic regression analysis model were applied to validate the phenotyping model. The HWE was determined by comparing the genotype frequencies with the expected values using the maximum likelihood method^58^. Differences between males and females regarding genotype frequencies were determined using a corrected χ^2^ test. To assess the effects of polymorphisms on the AFP pharmacokinetic parameters, comparisons between two and three groups were made. The Student’s *t*-test and one-way analysis of variance were used for parametric distributions, whereas Mann-Whitney U and Kruskal-Wallis H tests were used for nonparametric distributions. Post-hoc tests (Bonferroni correction and Tamhane’s T2 test) were used for pairwise comparisons. To confirm the contribution of genetic factors to the variability of pharmacokinetic parameters, automatic linear modelling using forward stepwise method, AICC and ASE as well as linear regression analysis using enter, stepwise, remove, backward, and forward methods were performed. Possible associations of genotypes or combinations of genotypes with phenotypes were evaluated using χ^2^ and Fisher’s exact tests and validated by logistic regression analysis. The evaluation effects of polymorphisms and associations were assessed under three different models (co-dominant, dominant, over-dominant, and recessive). The odds ratio (OR) was estimated with a 95% confidence interval (95% CI). All *P* values were two-tailed. Corrected *P* values (Pc) were obtained using the Bonferroni correction for exclusion of spurious associations. *P* < 0.05 was interpreted as statistically significant. The statistical analyses were performed with SPSS for Windows, V.20 (IBM Corp., NY, USA).

### Trial registration

Australian New Zealand Clinical Trials Registry: ACTRN12619000391178, date registered: 12/03/2019.

## Data Availability

All data generated and analysed during this study are included in this published article, but if necessary, some additional information is available from the corresponding author upon reasonable request.
